# Identification of *Bartonella* Trw Host-Specific Receptor on Erythrocytes

**DOI:** 10.1371/journal.pone.0041447

**Published:** 2012-07-26

**Authors:** Hon Kuan Deng, Danielle Le Rhun, Evelyne Le Naour, Sarah Bonnet, Muriel Vayssier-Taussat

**Affiliations:** 1 Unité Sous Contrat Bipar, French National Institute for Agricultural Research (INRA), Anses, Maisons-Alfort, France; 2 Key Laboratory for Zoonosis Research, Institute of Zoonosis, Jilin University, Changchun, People’s Republic of China; Instituto Butantan, Brazil

## Abstract

Each *Bartonella* species appears to be highly adapted to one or a limited number of reservoir hosts, in which it establishes long-lasting intraerythrocytic bacteremia as the hallmark of infection. Recently, we identified Trw as the bacterial system involved in recognition of erythrocytes according to their animal origin. The T4SS Trw is characterized by a multiprotein complex that spans the inner and outer bacterial membranes, and possesses a hypothetical pilus structure. *TrwJ, I, H* and *trwL* are present in variable copy numbers in different species and the multiple copies of *trwL* and *trwJ* in the *Bartonella trw* locus are considered to encode variant forms of surface-exposed pilus components. We therefore aimed to identify which of the candidate Trw pilus components were located on the bacterial surface and involved in adhesion to erythrocytes, together with their erythrocytic receptor. Using different technologies (electron microscopy, phage display, invasion inhibition assay, far western blot), we found that only TrwJ1 and TrwJ2 were expressed and localized at the cell surface of *B. birtlesii* and had the ability to bind to mouse erythrocytes, and that their receptor was band3, one of the major outer-membrane glycoproteins of erythrocytes, (anion exchanger). According to these results, we propose that the interaction between TrwJ1, TrwJ2 and band 3 leads to the critical host-specific adherence of *Bartonella* to its host cells, erythrocytes.

## Introduction

*Bartonella* species (*Bartonella* spp.) are small, curved, pleomorphic, fastidious, hemotropic, Gram-negative bacteria, mainly transmitted by arthropod vectors or via direct contact [1]. Until now, 24 species or subspecies, 13 of which being involved in human disease, have been formally validated [2]. Each of them appears to be highly adapted to a limited number of mammalian reservoir hosts, which results in relatively strict host specificity [1], [3].

*Bartonella* infection can cause many human and animal diseases. For example, *B. bacilliformis* causes Carrión’s disease, *B. quintana* causes trench fever and *B. henselae* causes a variety of clinical manifestations in humans: the main disease in immunocompetent individuals is cat scratch disease (CSD), whereas in immunocompromised patients it causes bacillary angiomatosis (BA) and bacillary peliosis (BP).

*Bartonella* spp., along with *Plasmodium spp.*, *Babesia spp.* and *Anaplasma marginale,* is one of the few infectious agents to infect erythrocytes [4]. The remarkableness, in contrast to other infectious agents infecting erythrocytes, is that all *Bartonella* spp. described to date, with the exception of the deadly *B. bacilliformis*, are maintained within the erythrocytes without having a significant effect on their physiology [5].

The dynamics of erythrocyte infection have been monitored in rats infected with fluorescently labelled *B. tribocorum*. After a primary phase, corresponding to the infection of a still unknown primary niche, potentially vascular endothelial cells [5], [6], [7], [8], [9], [10] or erythrocytic precursors [11]), *Bartonella* spp. reached the blood stream where they adhered to and invaded mature erythrocytes within 2 days. After infection, intracellular replication started immediately in a membrane-bound compartment, continuing over a period of several days until a steady number of intracellular bacteria was reached, the infected erythrocytes persisting in circulation for several weeks [5].

Bartonellae play an active role during erythrocyte invasion requiring both respiration and proton motive force [12], whereas treatment of erythrocytes with proton-motive force inhibitors has no effect on *Bartonella* adhesion. This suggests that erythrocytes play a passive role in invasion [13], [14], [15] and that *Bartonella* spp. are the main active participants in erythrocyte invasion.

The successful infection of a mammalian reservoir host erythrocyte by a *Bartonella sp.* typically involves a series of intimate host-pathogen interactions. On the molecular level this is reflected by attachment between *Bartonella* ligands and the erythrocyte receptors. The flagella of *B. bacilliformis* was identified to mediate initial erythrocyte adhesion [12]. This was supported by the reduction of the erythrocyte-binding ability of *B. bacilliformis* with anti-flagellin antibodies [16], and the poor adherence of non-motile variants and flagellin-minus mutant [17], [18]. Erythrocyte receptors for attachment to flagella have been partially characterized for *B. bacilliformis*. Buckles and McGinnis hill [19] demonstrated that *B. bacilliformis* was able to bind to several erythrocyte proteins: α and β subunits of spectrin, band 3 protein, glycophorin A, and glycophorin B. In addition, Iwaki-Egawa and Ihler [20] demonstrated that spectrin, actin and the other potential erythrocyte membrane proteins from different sources (human, cat, sheep) were able to bind to *B. bacilliformis* and *B. henselae*.

However, within the *Bartonella* genus, 13 *Bartonella spp.* are represented as a major phylogenic sub-branch of flagella-free *Bartonella*. All these flagella-free *Bartonella* possess a Trw Type 4 Secretion System (T4SS). T4SSs are supra-molecular transporters ancestrally related to bacterial conjugation [21]. In *Bartonella* spp., 2 T4SS, the VirB/D4 and Trw have been described and identified as pathogenicity factors required for bacterial colonization [22], [23]. Interestingly, the distribution of Trw and flagella among *Bartonella* spp. is mutually exclusive suggesting that, after its acquisition by horizontal transfer, the function of Trw evolved to replace that performed by flagella. In a recent study, using an *in vitro* model of erythrocyte adherence and invasion we demonstrated the direct role of Trw in erythrocyte recognition [23].

The *trw* genes of *Bartonella* species are collinear except for the presence of multiple tandem gene duplications of *trwL* and *trwJIH*. The multiple copies of *trwL* and *trwJ* are considered to encode variant forms of surface-exposed pilus components which are postulated to have a role in host-interaction with various surface structures of erythrocytes in different species. In contrast, the other duplicated genes, *trwI* and *trwH* are considered to encode the components required for pilus elongation and for pilus anchorage to the outer membrane, respectively [24].

Although the Trw locus has been identified as one of the *Bartonella* spp. factors involved in erythrocytic host-specific recognition, which of the Trw components are associated with the attachment, and the identity of the erythrocytic receptors are still unknown. In this study, combining different technologies and using the *B. birtlesii*/mouse erythrocytes model, we first identified that among the Trw components, only TrwJ1 and TrwJ2 were expressed at the bacterial surface and could bind to the erythrocyte membrane. Using Far Western blot we identified the major erythrocyte transmembrane glycoprotein Band3 as the receptor of the type IV TrwJ component.

## Materials and Methods

### Bacterial Strains and Growth Conditions

*Bartonella birtlesii* (*B. birtlesii*) (IBS 325^T^, CIP 106691^T^) were grown for 5 days on Columbia agar containing 5% defibrinated sheep blood (CBA) in a humidified atmosphere with 5% CO2 at 35°C.

*E.coli* TOP10 (Invitrogen, USA), BL21 Star (Invitrogen, USA) and BL21 pLysS (Novagen, Germany) were grown overnight in Luria-Bertani (LB) broth or on LB agar plates supplemented when needed with carbenicillin (50 µg/mL) at 37°C.

### Animals and Ethics Statement

Animals were handled in strict accordance with good animal practice as defined by the relevant European animal welfare body. Animal work was approved by our institute’s ethics committee. The protocol was approved by the Committee on the Ethics of Animal Experiments of the National Veterinary School of Alfort (Permit Number: 2008-11).

Six-week old OF1 or Balb/C female mice were housed in an animal facility (5 mice per cage) for blood sampling or immunization with recombinant proteins. Cats used for blood sampling came from the National Veterinary School of Alfort. White New Zealand male rabbits (16 weeks old) were used to produce polyclonal antibodies against murine band 3 extracted from erythrocytic membrane.

### Isolation of Erythrocytes

Erythrocytes from the peripheral blood of mice and cats were isolated and purified by Ficoll gradient centrifugation as previously described [25]. After washing in PBS, erythrocytes were maintained in F12 modified medium (F12 medium supplemented with 10% foetal calf serum, 2 mM glutamine, 1 mM sodium pyruvate, 0.1 mM Hepes, 257 mM Histidine (His), 0.1 mg/ml Hematin/His, and non-essential amino acid) (Gibco, France) before being used for further analysis (erythrocyte invasion assay, phage binding assay).

### Trw Proteins Expression and Purification

Genomic DNA was isolated from *B.birtlesii* using the Roche high pure PCR template preparation kit (Roche, Switzerland). Based on the entire *trwJ1, trwJ2, trwL1, trwL2,trwL3, trwL4* and *trwL5* sequences (F. Biville, unpublished data), DNA inserts corresponding to *trw* genes were amplified by PCR from genomic *B. birtlesii* as template and the corresponding specific primers (shown in table 1). PCR consisted of an initial denaturation step at 98°C for 2 min followed by 30 cycles of denaturation at 98°C for 20 s, annealing at 55°C for 50 s and extension at 72°C for 50 s, and a final extension step at 72°C for 10 min. All PCR reactions were performed in a MyCycler™ thermocycler (Biorad, USA) with the Phusion high-fidelity DNA polymerase (New England Biolabs, USA).

PCR products were ligated to the PET-102 expression vector (Invitrogen, USA). This vector allows expression of recombinant protein containing a thioredoxin epitope followed by an enterokinase recognition site at the N-terminal end and a 6-His tag at the C-terminal end. After propagation of the recombinant plasmids in *E.coli* TOP10, they were then transformed into BL21 Star and BL21 pLysS by electroporation. Expression was obtained for *trwJ1* and *trwJ2* in *BL21 Star* incubated with 0.5 mM IPTG (isopropyl β-D-thiogalactoside) for 4 hours at 22°C, and for *trwL2*, *trwL3*, *trwL4*, and *trwL5* in *BL21 pLysS* incubated with 0.5 mM IPTG for 4 h at 37°C.

The recombinant fusion proteins were purified by affinity chromatography using the nickel-nitrilotriacetic acid (Ni-NTA) resin following the manufacturer’s protocol (Qiagen, Germany) under native conditions or denaturing conditions according to their properties. For mice immunization, the thioredoxin parts of the recombinant proteins were cut off by enterokinase (Invitrogen, USA). The digestion reactions were performed in the Ni-NTA-protein mixture overnight at 37°C, under shaking, in 1 ml containing 10X enterokinase buffer and 25U of enterokinase and were followed by 3 washes in PBS. In each case the recombinant proteins were eluted from the resin in 400 µl native elution buffer (300 mM NaCl, 50 mM NaH2PO4, 250 mM imidazole, pH 8.0) or denaturing elution buffer (8 M Urea, 300 mM NaCl, 50 mM NaH2PO4, 250 mM imidazole, pH 8.0).

The purified recombinant proteins were analyzed by a 15% SDS-polyacrylamide gel electrophoresis (SDS-PAGE), followed by gel staining with Coomassie brilliant blue R-250 (Sigma, USA). The SigmaMarker™ low range (Sigma; USA) was used as reference for the molecular weights.

### Production of Murine Polyclonal Antibodies against Recombinant Trw Proteins

Balb/C mice were injected twice subcutaneously with 10 µg of each recombinant protein mixed in oil Montanide® adjuvant ISA-70 (Seppic, France) at 2-weeks-interval with the same antigen dose. Sera were collected 15 days after the last immunization and stored at -20°C. The titres of polyclonal antibodies were determined by dot-blot analysis using the corresponding recombinant proteins.

### Western Blot (WB) Analyses

*B. birtlesii* (1.10^8^ UFC from 5 days growth on CBA plates) and 0.1 µg of rTrwJ1, rTrwJ2, rTrwL2, rTrwL3, rTrwL4, rTrwL5 recombinant proteins were reduced with 100 mM DTT, resolved by a Tris-Glycine 15% SDS-PAGE gel and blotted onto PVDF membranes (GE Healthcare, UK) at 15 V for 12 min by Trans-Blot® SD Semi-Dry Electrophoretic Transfer Cell Instruction (Biorad, USA) in Towbin transfer buffer (25 mM Tris, 192 mM glycine, 20% methanol, pH 8.3). The PVDF membranes were blocked in 1X blocking buffer (50 mM Tris, 150 mM NaCl pH 7.4 and 0.05% Tween-20, 5% non-fat dried milk) for 1 h at 37°C and then incubated with 1/1000 dilution of mouse anti-Trw proteins polyclonal antibodies for 1 h at 37°C in blocking buffer. Anti-Trw labelling assays were revealed with an anti-mouse IgG (H+L) alkaline phosphatase (AP)-goat antibody (1∶10,000; Jackson ImmunoResearch Laboratories, USA) for 1 h at 37°C, and a 10 ml solution of NBT (Nitro blue tetrazolium chloride)/BICP (5-Bromo-4-chloro-3-indolyl-phosphate p-toluidine salt) (Sigma, Germany).

### Electron Microscopy and Immunolocalization of Trw Components

Pellets of bacteria were fixed for 30 min with 2% paraformaldehyde solution in PBS, then centrifuged and washed in PBS. The bacteria were collected onto 400 mesh formvar-coated nickel grids. Grids were quenched with NH4Cl 50 mM in PBS, blocked with PBS containing 1% BSA, and 0.1% BSA-c™ (BioValley, France). Antibodies (anti-TrwJ1, anti-TrwJ2, anti-TrwL2-L5, naïve mouse serum) were added at a 1/100 dilution in PBS containing 1% BSA, 0.1% BSA-c™ and incubated over night at +4°C. The grids were then washed twice for 3 min in PBS -1% BSA, 0.1% BSA-c™ and goat anti-mouse IgG (1/50 dilution) coupled to 10 nm colloidal gold particles (British Biocell International – TEBU, France) added for 1 hour. The grids were again washed twice with PBS -BSA, twice with PBS, and fixed for 5 min with 2.5% glutaraldehyde in PBS. Finally, the grids were washed three times with distilled water and air dried.

The grids were then examined with a Zeiss EM902 electron microscope operated at 80 kV (Carl Zeiss – France), and images were acquired with a charge-coupled device camera (Megaview III) and analysed with ITEM Software (Eloïse, France) MIMA2 Platform, INRA-CRJ (http//MIMA2@jouy.inra.fr).

### Expression of TrwJ1 and TrwJ2 on T7 Phage

The T7 select 10-3b Cloning kit (Novagen, Germany) containing the T7 select 10-3b *EcoRI*/*HindIII* vectors and T7 packaging extracts was used to display TrwJ1 and TrwJ2 on T7 phage. Briefly, *trwJ1* and *trwJ2* genes were amplified by PCR using the specific primers described in table 1. To allow insertion in T7 phage sequences, the forward primer contained an *EcoRI* restriction enzyme site while the reverse primer contained a *HindIII* restriction enzyme site.

After PCR amplification, the PCR products were digested by *EcoRI* (TaKaRa, Japan) and *HindIII* (TaKaRa, Japan) and purified by PCR clean-up Gel extraction Kit (MACHEREY-NAGEL, Germany), before being packaged, titered and amplified following the procedures outlined in T7Select system.

### Phage Binding Assay with Mouse and Cat Erythrocytes

Mouse and cat erythrocytes were resuspended in PBS at 1×10^8^ cells/ml, and incubated with 1×10^9^ PFU TrwJ1-T7 or TrwJ2-T7 phages with shaking for 4 hours at 35°C. The bound phages were separated as previously described with slight modification [26]. Briefly, 300 µl of the cell-phage mixtures were gently transferred to the top of a non-miscible dibutyl phthalate/cyclohexane (9∶1 [v:v]) organic lower phase (600 µl) and centrifuged at 10,000 g for 10 min. The supernatants were drawn-off. Bound phages were eluted from cells for 10 minutes at room temperature by adding 500 µl of 1% SDS. The titres of the bound phages were then determined by following the procedures outlined in T7Select system.

### Effect of Anti-Trw Antibodies on *in vitro* Infection of Mouse Erythrocytes by *B. birtlesii*

The effect of the different mouse anti-Trw antibodies on the invasion capacity of erythrocytes by *B. birtlesii* was measured *in vitro* as described [23]. Briefly, after culturing *B. birtlesii* for 5 days on CBA plates, the bacteria were harvested, washed in PBS and suspended in F12 modified medium. Anti-Trw antibodies (1/100 dilution) or serum from a non-immunized mouse (1/100 dilution) were then incubated with bacteria at 35°C for 4 h, while the control was incubated with F12 modified medium. In each case, bacteria were then added to mouse erythrocytes at a multiplicity of infection (MOI, calculation based on 1 OD_600_ nm = 3×10^9^ bacteria/ml) of 1 and incubated at 35°C. After 48 h of invasion, the erythrocytes were separated from the non-associated bacteria by washing 3 times with PBS and centrifuged at 1500 rpm for 10 min. The erythrocytes were then incubated with 50 µl gentamicin sulfate (125 µg/ml) for 2 h at 35°C to kill any residual extracellular bacteria, washed three times in PBS to remove the antibiotic and then any intracellular bacteria were released by hypotonic lyses of the erythrocytes in 20 µl of sterile water by freezing at -20°C for 15 min. After thawing, serial dilutions of bacteria in PBS were inoculated onto CBA plates and incubated for 5 days before being counted. The impact of anti-Trw antibodies on invasion capacity was then evaluated by comparing the numbers of intra-erythrocytic bacteria with or without antibodies.

### Identification of TrwJ2 Erythrocytic Receptor by Far-Western Blot

Far Western blot aims to detect interactions between two proteins, in our case TrwJ2 and receptor. Briefly, this technique consists in (1) performing SDS-PAGE of erythrocytic membrane protein, containing potential receptor (2) fixing receptor to the membrane by transfer (3) Incubating the membrane with the recombinant and purified TrwJ2 and (4) Revealing with antibodies against putative receptor, band 3.

Lysates of erythrocytic membranes were prepared from frozen blood (5×10^9^ erythrocytes) samples, thawed, resuspended in stabilization solution (ID-CellStab, Diamed) and washed in 0.9% NaCl (B. Braun Medical). Membranes were prepared at 0–4°C by hypotonic lysis with 5P8 buffer (5 mM Na_2_HPO_4_, pH 8.0 and 350 µM EDTA, pH 8.0), stripped by incubation with 10 mM NaOH and finally solubilized with an equal volume of 4X LDS Sample buffer (Invitrogen). Erythrocytic membrane lysates were reduced with 100 mM DTT, resolved by Tris-Glycine 8% SDS-PAGE and transferred onto PVDF membranes at 20 V for 25 min by Trans-Blot® SD Semi-Dry Electrophoretic Transfer Cell. The PVDF membranes were blocked in 1X blocking buffer and incubated for 2 hours at RT with 200µg of rTrwJ2 in 10 ml blocking buffer, immunodetected with 1/1000 dilution of anti-TrwJ2 polyclonal antibodies and 1/10,000 dilution of AP-goat anti-mouse IgG (H+L) (1/10,000), then stained with a solution of NBT/BICP as above. The PVDF membranes were similarly reprobed with a 1/100 dilution of anti-band3 (C-17) monoclonal antibodies (Santa Cruz Biotechnology, USA) and a 1/30,000 dilution of AP-goat anti-donkey IgG (Jackson ImmunoResearch Laboratories, USA), and stained with a solution of NBT/BICP as above.

### Inhibition of *Bartonella*-erythrocytes Interaction using Anti-band 3 Polyclonal Antibodies

As commercially available anti-band3 antibodies did not recognize all the surface part of band3, polyclonal antibodies raised against the entire sequence of murine erythrocytic band3 were produced as follows: lysates of erythrocytic membrane were resolved by Tris-Glycine 8% SDS-PAGE gels, the band corresponding to the size of band3 (90 kDa) was cut from the gels, grinded and resuspended in PBS.

Two rabbits were injected subcutaneously with 200µg of murine erythrocytic band 3 mixed in Montanide® oil adjuvant ISA-70. Second and third injections were given at 2-week-interval with the same antigen dose. Sera were collected 15 days after the final immunization and stored at -20°C. The titres of polyclonal antibodies were determined by dot-blot analysis using the purified erythrocytic band3 protein.

The effect of anti-band3 polyclonal antibodies on the interaction between TrwJ1-T7 or TrwJ2-T7 phages and mouse erythrocyte was assessed by incubating 1×10^8^ mouse erythrocytes with anti-band3 antibodies (1/100 dilution) or serum from a non-immunized rabbit (1/100 dilution) at 35°C for 4 h, while the control was incubated with PBS. Then 1×10^9^ PFU of TrwJ1-T7 or TrwJ2-T7 phages were added and phage binding assays with mouse erythrocytes were performed as described above.

In parallel, the effect of anti-band3 polyclonal antibodies on the invasion capacity of erythrocytes by *B. birtlesii* was measured by incubating anti-band3 antibodies (1/150 dilution) or serum from a non-immunized rabbit (1/150 dilution) in *B. birtlesii-*erythrocyte mixture at 35°C for 4 h, while the control was incubated with F12 modified medium. The intracellular bacteria were quantified as described above.

## Results

### 1- Identification of Trw Components that are Expressed at the *B. birtlesii* Cell Surface

Candidate genes for mediating Trw interaction with erythrocytic receptors encoded surface-exposed components. Among the Trw components, the T4SS pilus components TrwL (L1 to L5) and TrwJ (J1 and J2) were shown to be putative surface proteins [27]. We checked whether they were indeed expressed at the *B. birtlesii* surface by first producing polyclonal antibodies which reacted specifically with each of the corresponding recombinant proteins.

Recombinant soluble proteins rTrwJ2, rTrwL2, rTrwL3, rTrwL4 and rTrwL5 were expressed and recovered from the supernatant of lysated *E. coli*, while recombinant rTrwJ1 was recovered as an insoluble form in the inclusion body of *E. coli*. Despite many assays using different *E. coli* strains and different culture conditions, we failed to express rTrwL1.

**Figure 1:**
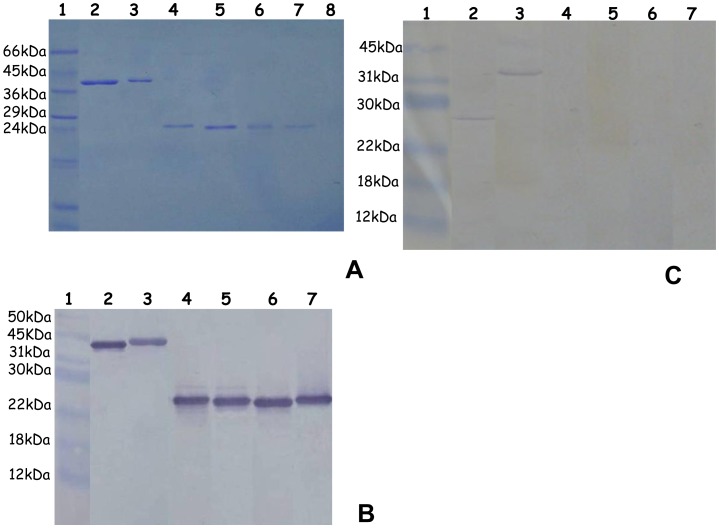
Expression and detection of the putative surface Trw components. A - SDS electrophoresis of the purified recombinant Trw proteins. SDS-PAGE analysis under reduced condition of Trw recombinant proteins expressed in *E. coli* after purification by affinity chromatography and without elimination of the thioredoxin epitope. Lane 1, Low range molecular weight (Sigma); Lane 2, rTrwJ2; Lane 3, rTrwJ1; Lane 4, rTrwL5; Lane 5, rTrwL4; Lane 6, rTrwL3; lane 7, rTrwL2; Lane 8, rTrwL1. B - Western blot detection of the purified recombinant Trw proteins Immunoblot analysis of Trw recombinant proteins expressed in *E. coli* after purification by affinity chromatography, without elimination of the thioredoxin epitope, and after separation on SDS-PAGE under reduced conditions, using polyclonal antibodies against rTrwJ2 (lane 2), rTrwJ1 (lane 3), rTrwL5 (lane 4), rTrwL4 (lane 5), rTrwL3 (lane 6), rTrwL2 (lane 7). Lane 1, Prestained molecular weight marker (New England Biolabs). C - Western blot detection of the *B. birtlesii* Trw proteins Western blot detection of total *B.birtlesii* proteins separated by SDS-PAGE under reduced conditions, using polyclonal antibodies against rTrwJ1 (lane 2), rTrwJ2 (lane 3), rTrwL2 (lane 4), rTrwL3 (lane 5), rTrwL4 (lane 6) and rTrwL5 (lane 7). Lane 1, Prestained molecular weight marker (New England Biolabs).

After purification, a single band corresponding to each purified recombinant protein was identified on SDS-PAGE on a Coomassie stained acrylamide gel. The observed molecular mass corresponded to the predicted size of the recombinant protein with the addition of 13 kDa corresponding to the thioredoxin motif and 3 kDa corresponding to the V5 and 6×His-tag motifs, i.e. 43.5 kDa for rTrwJ1, 42 kDa for rTrwJ2, 23.5 kDa for rTrwL2, 23.5 kDa for rTrwL3, 23.5 kDa for rTrwL4 and 23.5 kDa for rTrwL5 (Figure 1A).

The thioredoxin-free recombinant proteins were used to produce polyclonal antibodies from immunized Balb/C mice. The obtained polyclonal antibodies reacted with the corresponding recombinant proteins as shown in Figure 1B.

Recognition of native proteins by antibodies was then evaluated by western blot on proteins extracted from *B. birtlesii* culture on agar plates and separated on SDS-PAGE. As shown in Figure 1C, only TrwJ1 and TrwJ2 were detected, while Trw L2, L3, L4, L5 were not (Figure 1C). The molecular mass observed for TrwJ1 corresponded approximately to the one calculated from the sequence (27.5 kDa). On the contrary, the molecular mass observed for TrwJ2 was higher than was expected from the sequence (26 kDa) with signal peptides around 32 kDa. This difference would suggest the presence of aggregates or post-translational modifications.

**Figure 2:**
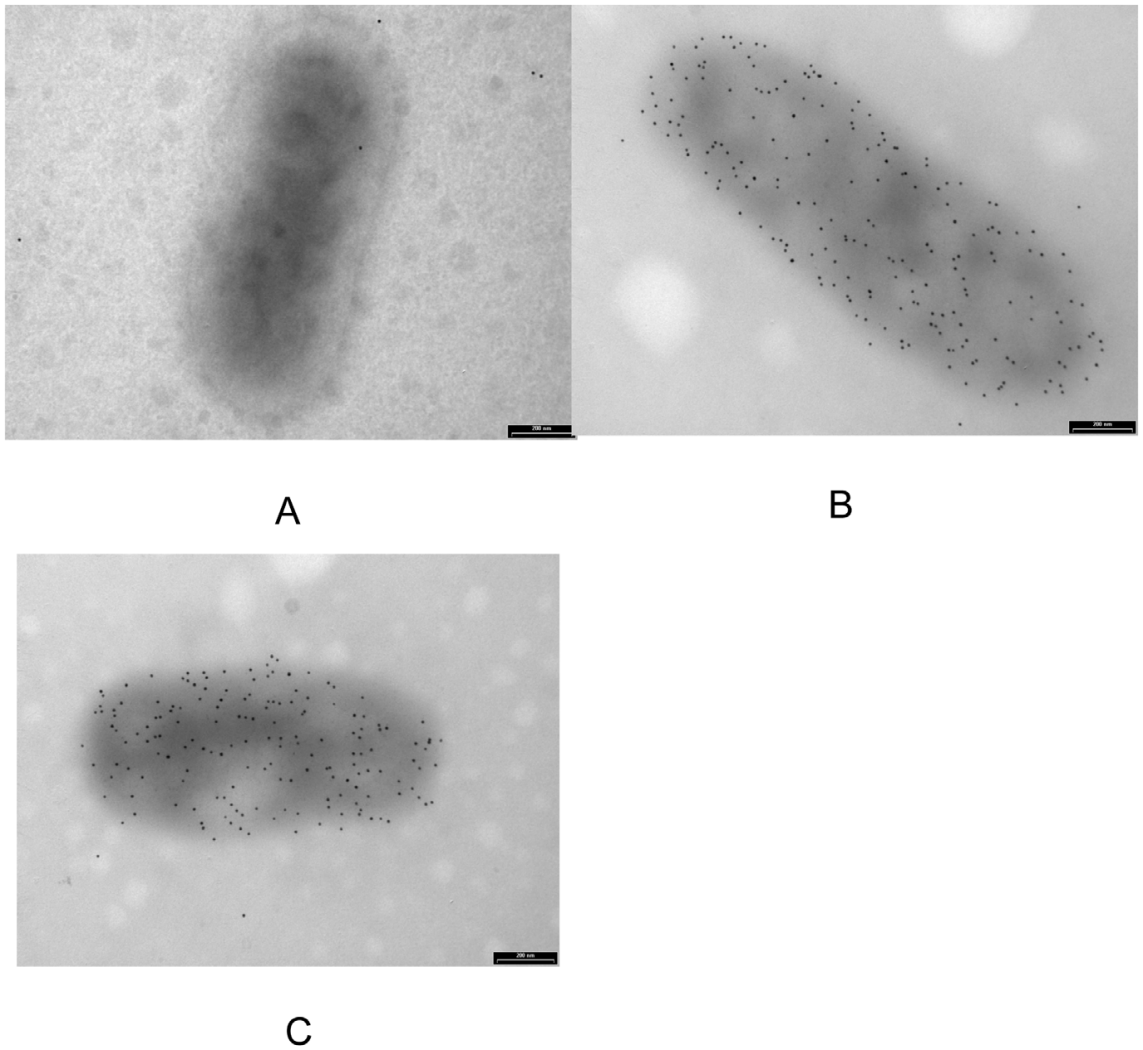
Immunogold labelling and transmission electron microscopy of Trw components on the *B. birtlesii* surface. Electron microscopy detection of TrwJ1 and TrwJ2 on the surface of CBA-cultivated *B. birtlesii* using polyclonal antibodies against rTrwJ1 (B) and rTrwJ2 (C) recombinant proteins. (A), naïve mouse serum as negative control.

The different putative Trw surface components were localized by immunostaining *B. birtlesii* whole bacteria with the different anti-Trw polyclonal antibodies and electron microscopy observations. TrwJ1 and TrwJ2 were detected at the *B. birtlesii* cell surface whereas none of the TrwL proteins was detected at the cell surface (Figure 2).

### 2– TrwJ1 and TrwJ2 Interact with Mouse Erythrocytes

**Figure 3:**
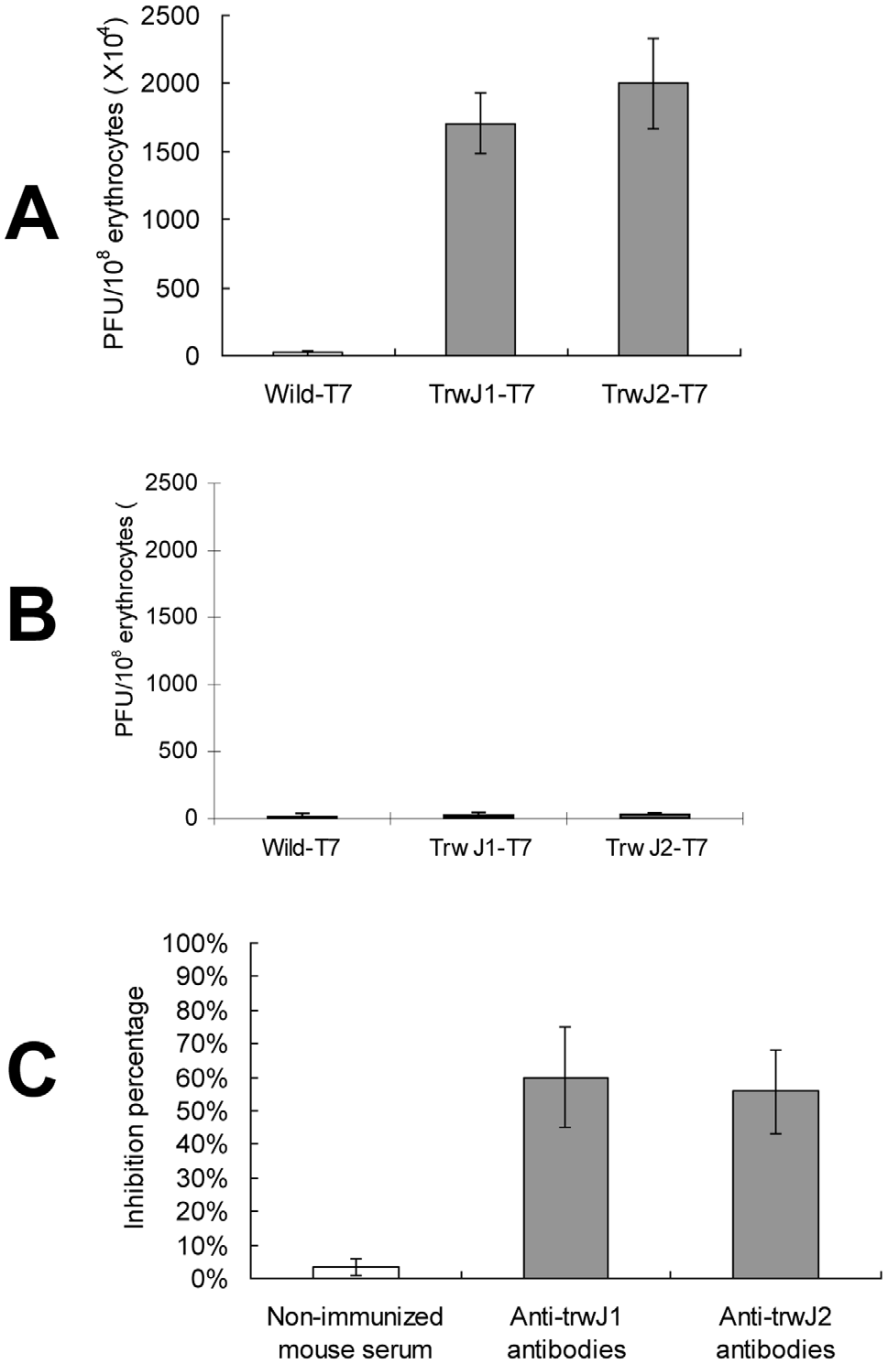
Identification of TrwJ1 and TrwJ2 interaction with mouse erythrocytes. A – Binding assay between mouse erythrocytes and T7 fusion phages Evaluations of the capacity of TrwJ1-T7 and TrwJ2-T7 phages to bind to mouse erythrocytes were evaluated three times (3 replicates each). Wild-T7 phages were used as control and the values are presented as the mean of three independent experiments. B – Binding assay between cats erythrocytes and T7 fusion phages Evaluation of the capacity of TrwJ1-T7 and TrwJ2-T7 phages to bind to cat erythrocytes were evaluated once in 3 replicates each. Wild-T7 phages were used as control and the values are presented as the mean of three experiments. C – Efficiency of *in vitro* invasion inhibition of mouse erythrocyte by *B. birtlesii* with anti-Trw polyclonal antibodies Invasion inhibition assays with polyclonal antibodies against rTrwJ1 and rTrwJ2 recombinant proteins and serum from non-immunized mouse were performed three times (3 replicates each). The values are presented as the mean of three independent experiments.

Two complementary analyses were performed to see whether TrwJ1 and TrwJ2 were able to bind to mouse erythrocytes: the first analysis consisted of measuring the capacity of TrwJ1-T7 as well as TrwJ2-T7 phages to bind to mouse or cat erythrocytes; the second consisted of evaluating the capacity of anti-TrwJ1 and anti-TrwJ2 polyclonal antibodies to inhibit mouse erythrocyte invasion by *B. birtlesii.* As shown in Figures 3A and 3B, T7 phage displaying *B. birtlesii* TrwJ1 and TrwJ2 were able to bind to mouse erythrocytes but not to cat erythrocyte. The amounts of TrwJ1-T7 and TrwJ2-T7 phages which were able to bind to 1×10^8^ mouse erythrocytes were 1.7×10^7^ PFU and 2×10^7^ PFU respectively, while wild-T7 phages were unable to bind to mouse erythrocytes. Addition of anti-TrwJ1 and anti-TrwJ2 polyclonal antibodies to the *B. birtlesii*-erythrocytes invasion mixture, significantly reduced the invasion of mouse erythrocytes by *B. birtlesii* by 60% and 55.7% respectively, while serum from a non-immunized mouse did not reduce invasion (Figure 3C).

### 3 Identification of Erythrocytic Receptor of TrwJ2

**Figure 4:**
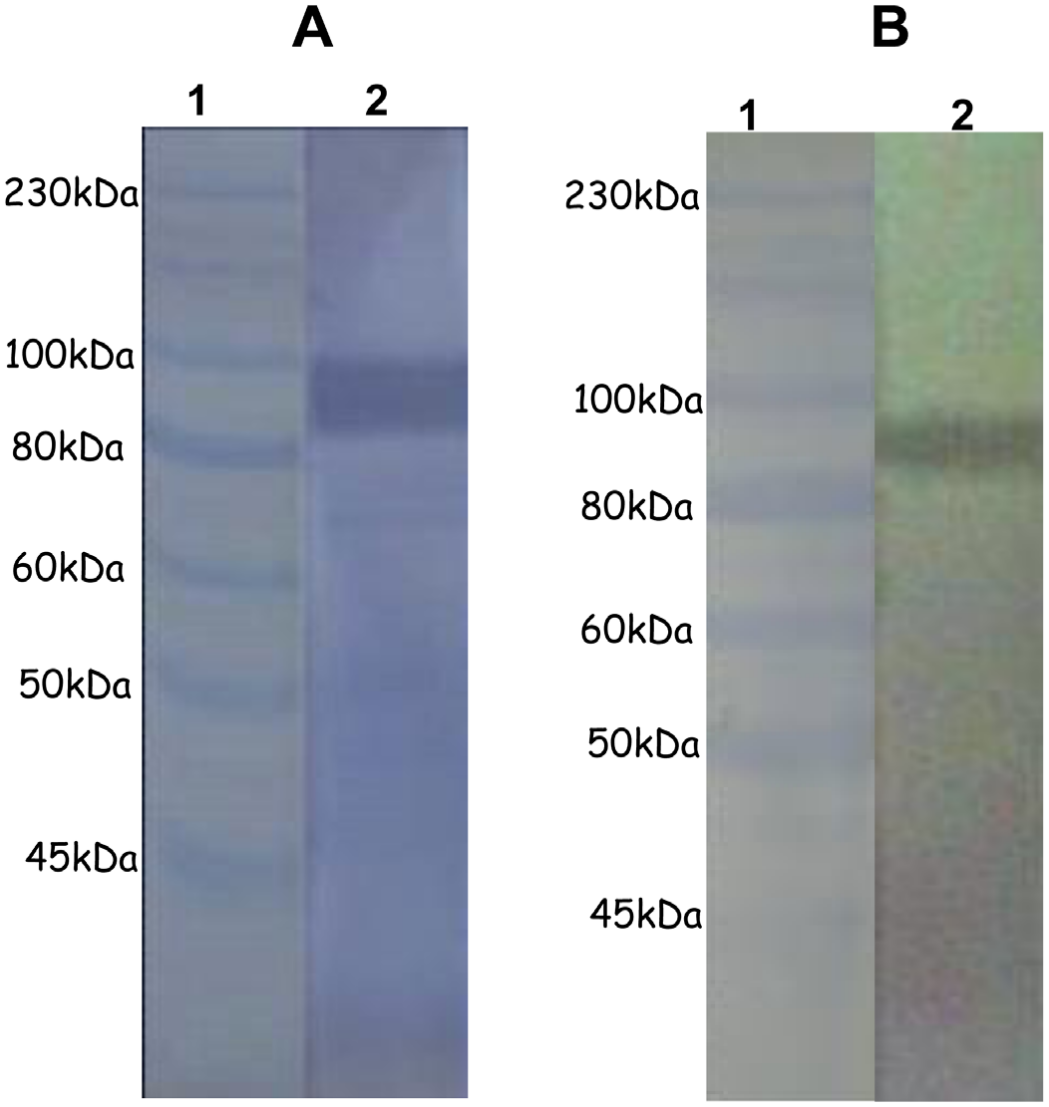
Identification of TrwJ2 erythrocytic receptor. A – Far Western blot analysis of mouse erythrocyte membrane proteins using rTrwJ2 recombinant protein Mouse erythrocyte membrane proteins were separated on SDS-PAGE under reduced conditions, transferred (on? to?) PVDF membrane, probed with rTrwJ2, and analysed by immunoblot using anti-mouse antibodies (lane 2). Lane 1, prestained molecular weight marker (New England Biolabs). The experiment was conducted twice with qualitatively similar results. B –Immunoblot analysis of mouse erythrocytic membrane proteins using Band3 monoclonal antibody Mouse erythrocyte membrane proteins were separated on SDS-PAGE under reduced conditions, transferred (on? to?) a PVDF membrane, and analysed by immunoblot using anti-band3 antibodies (lane 2). Lane 1, Prestained molecular weight marker (New England Biolabs).

As TrwJ1 was expressed as an insoluble form, identification of the receptor was only conducted with TrwJ2 by Far-Western blotting. When recombinant rTrwJ2 is incubated with mouse erythrocytic membrane proteins, antibodies against TrwJ2 react with a single band which corresponds to a mouse erythrocytic membrane protein with an estimated size of 90 kDa (Figure 4A). This band corresponds to the size of the erythrocytic band3 as validated by immunoblot with goat anti-band3 monoclonal antibody (Figure 4B).

**Figure 5:**
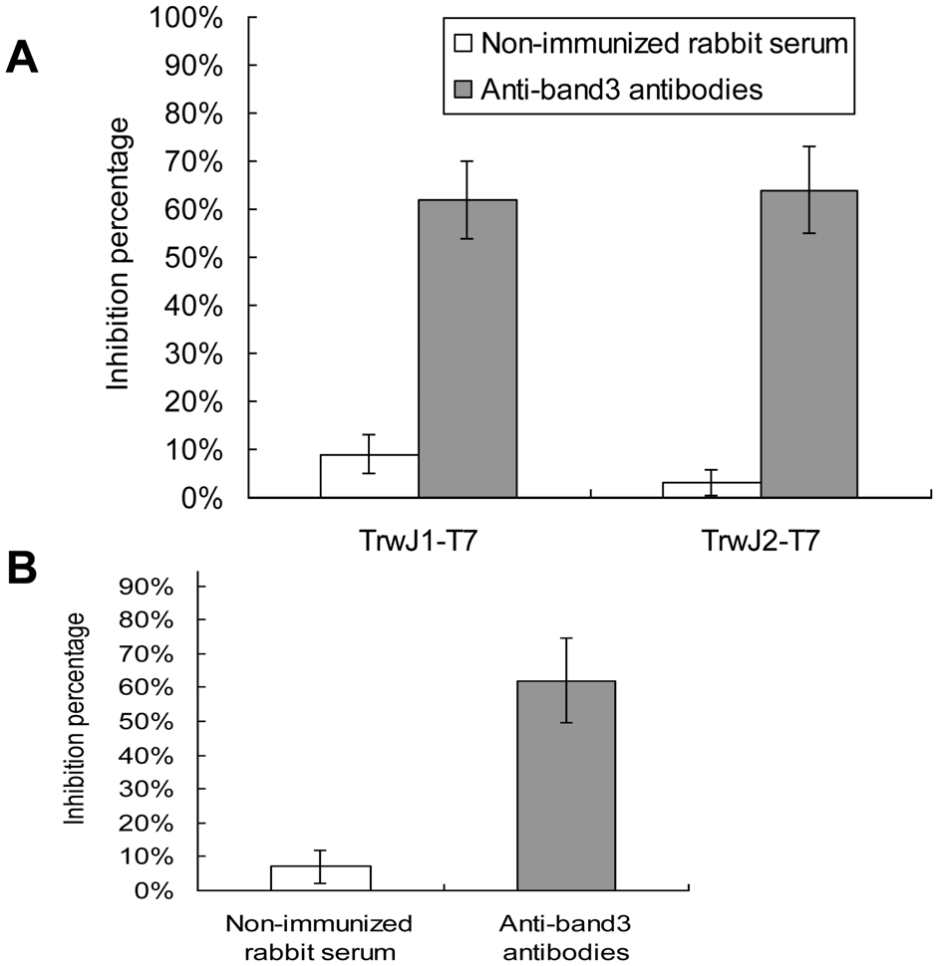
Role of erythrocytic band3 in adhesion and invasion between mouse erythrocyte and *B. birtlesii*. A – Analysis of the impact of anti-band3 antibodies on the interaction between mouse erythrocytes and TrwJ1-T7 or TrwJ2-T7 phages. Phage binding inhibition assays with anti-band3 polyclonal antibodies and serum from non immunized rabbit were performed three times (3 replicates each).The values are presented as the mean of three independent experiments. B – Analysis of the impact of anti-band3 antibodies on the invasion capacity of mouse erythrocyte by *B. birtlesii* Invasion inhibition assays with anti-band3 polyclonal antibodies and serum from non immunized rabbit were performed three times (3 replicates each). The values are presented as the mean of three independent experiments.

To further demonstrate that band 3 was or was not a receptor of *B. birtlesii* TrwJ, we then evaluated the capacity of rabbit anti-band3 polyclonal antibodies to inhibit erythrocyte binding with *B. birtlesii* TrwJ1-T7 or TrwJ2-T7 phages and erythrocyte invasion by *B. birtlesii*. As shown in Figures 5A and 5B, adding anti-band3 polyclonal antibodies to the TrwJ1-T7 or TrwJ2-T7 phages-erythrocytes binding mixture significantly reduced phage binding capacity by 62% and 64% respectively, and significantly reduced *B. birtlesii* invasion capacity by 62%, while serum from non-immunized rabbit had only a very slight influence on both adherence and invasion.

## Discussion

Bacteria-specific adhesion to host cells can be defined as the selective binding between a specific molecular component on the bacterial surface and a substratum-specific receptor in the host cells. We have previously shown that in Bartonella species, the T4SS Trw is involved in erythrocytic recognition [23]. T4SS Trw is characterized by a multiprotein complex that spans the inner and outer bacterial membranes, and possesses a hypothetical pilus structure. TrwJ and trwL are thought to encode minor and major pilus components, which are considered to be potentially responsible for the interaction with erythrocyte [27]. The aim of the present study was therefore to evaluate the role of TrwJ and TrwL proteins in adhesion to erythrocytes.

*B.birtlesii trwJ1*, *trwJ2*, *trwL2*, *trwL3*, *trwL4* and *trwL5* were expressed as recombinant proteins and polyclonal antibodies against these proteins were produced, firstly to estimate their expression and secondly to localize them on the cell surface of the bacteria. Immunoblot analysis showed that only TrwJ1 and TrwJ2 could be detected on CBA-cultivated *B. birtlesii*, and not TrwL2, L3, L4, or L5, suggesting that these latter were not expressed in *in vitro* culture, or at an undetectable level. Some studies showed that homologues of TrwJ (VirB5) and TrwL (VirB2) were not detected in wild and complemented *Agrobacterium tumefaciens*, whereas they could be detected in induced complemented cells [28], [29], [30], [31]. Similarly the homologue of VirB5 was not detected in *B. henselae* cultivated on cell-free laboratory medium but could be detected in bacteria incubated with HMEC-1 cells [32]. The Trw T4SS has also been identified as being upregulated intracellularly during *B. henselae* interaction with HUVECs or ECs [8]. Finally, *Bartonella* has the ability to infect different hosts (reservoir or incidental mammalian as well as arthropod hosts), and different host cell types, which suggests the existence of different pathogenicity factors on its surface that are presumably controlled by differential gene expression during the course of infection. These findings suggest that the expression of TrwL, like that of its homologues in other bacteria, might be regulated in response to infection signals, although further work is necessary to unravel the molecular details of this mechanism.

Results obtained by immunoblot analysis were then confirmed by electron microscopic analysis as only Trw J1 and TrwJ2 were detected at the cell surface of *B. birtlesii*. For these reasons, TrwJ1 and TrwJ2 appeared to us as the best potential candidates for *in vitro* interaction between *B. birtlesii* and erythrocytes.

We then investigated whether the surface Trw components TrwJ1 and TrwJ2 were associated with the adherence to erythrocytes, by constructing phage displaying *B. birtlesii* TrwJ1 and TrwJ2. The results showed that *B. birtlesii* TrwJ1-T7 and TrwJ2-T7 phages were able to bind to mouse erythrocytes, while wild-T7 phages showed significantly less binding ability. We then confirmed this result by evaluating the capacity of anti-TrwJ1 and anti-TrwJ2 polyclonal antibodies to inhibit mouse erythrocyte invasion by *B. birtlesii.* We found that incubation with both polyclonal antibodies resulted in inhibition of the invasion of mouse erythrocytes by *B. birtlesii.* These results clearly suggest that TrwJ1 and TrwJ2 are associated with adherence of the bacteria to erythrocytes. As we have previously shown that the Trw T4SS of *Bartonella* mediates host-specific adhesion to erythrocytes, and that *B. birtlesii* is unable to bind and invade cat erythrocytes [23], we performed the same experiment, using cat erythrocytes and showed that *B. birtlesii* TrwJ1-T7 and TrwJ2-T7 phages were not able to bind to cat erythrocytes. These results enlarged on those obtained in earlier studies of the relationship between Trw and the host erythrocyte, and now suggest that this host-specific adhesion is mediated by TrwJ1 and TrwJ2.

Although TrwL were not detected on the surface of bacteria, this does not exclude an interactive role between the bacteria and their host cells. Indeed, after adherence, bacteria use other virulence factors to become more intimately bound to their host cells via specific and stable interactions that can mediate invasion [33]. Mutagenesis of TrwL is reported to lead to inhibition of intraerythrocytic bacteremia in the reservoirs of for *B. tribocorum* and *B. birtlesii* [22], [23], and to loss of the capacity of *B. birtlesii* to infect mouse erythrocyte *in vitro* [23] thus demonstrating that TrwL also has an essential role in erythrocyte invasion, both *in vivo* and *in vitro*. However, in the absence of direct proof, to suggest that TrwL is involved in intimate adhesion rather than in the initial adhesion occurring during infection of the erythrocytes, remains speculative.

By conducting experiments to determine the receptor of TrwJ1 and TrwJ2, we found that TrwJ2 recombinant protein was able to bind a major glycoprotein present in mouse erythrocyte membrane: band3. We also demonstrated that, *in vitro*, polyclonal antibodies raised against mouse Band-3 were able to inhibit the adhesion between TrwJ1-T7 and TrwJ2-T7 phages and mouse erythrocytes and reduce the mouse erythrocyte invasion capacity of *B. birtlesii*. Taken together, all these results clearly suggest an interaction between TrwJ1, TrwJ2 and Band 3 leading to critical adherence of the bacteria to its host cells, the erythrocytes.

Band 3 is a major transmembrane glycoprotein of the erythrocyte membrane and functions in anion transport [34]. It has been suggested to be one of the possible erythrocyte receptors of *B. bacilliformis* [19]. Erythrocytic band 3 has also been suggested to be involved in the malaria parasite invasion of erythrocytes [35], [36], [37], [38], [39], [40]. In addition, recent studies have revealed that *P. falciparum* merozoite surface protein 1 (MSP1), an essential parasite protein has a conserved role in the invasion of erythrocytes by *P. falciparum* and *P. chabaudi* [41], [42] and this protein interacts with two nonglycosylated exofacial regions of erythrocyte band 3, designated 5ABC (amino acids 720–761) and 6A (amino acids 807–826) [43]. Two regions of merozoite surface protein 9 (MSP9), which is also known as an acidic basic repeat antigen, interact directly with 5ABC during erythrocyte invasion by *P. falciparum* [44], [45]. Erythrocyte invasion by *P. falciparum* is thought to proceed via two distinct pathways [46], [47]: a sialic acid-dependent pathway mediated by glycophorin A, B and C [48], [49], [50], [51], [52], and a sialic acid-independent pathway mediated by band3, as described above. Concerning *Bartonella*, a previous study showed that pre-treatment of feline erythrocytes with neuraminidase and trypsin had no effect on *B. henselae* invasion, indicating that invasion occurs via a sialic acid-independent pathway [53]. As we have identified band 3 as the erythrocyte receptor of *Bartonella*, we attempted to determine whether or not the sialic acid-dependent erythrocyte receptors of *P. falciparum* were also involved in *Bartonella* infection. Preliminary results demonstrated that the anti-mouse N-terminal extracellular domain of glycophorin A polyclonal antibodies reduced invasion of mouse erythrocytes by *B. birtlesii* by approximately 50% (data not shown). This result provides additional information which allows us to hypothesize that *Bartonella*-erythrocyte interactions may also be mediated by two distinct pathways, and expands our understanding of the biology and infection course of *Bartonella* spp., which is still far from completely understood. Further studies are now conducted to elucidate the complete mechanisms involved in erythrocyte invasion by *Bartonella* spp.
